# Genotyping of *Plasmodium vivax* by minisatellite marker and its application in differentiating relapse and new infection

**DOI:** 10.1186/s12936-016-1139-3

**Published:** 2016-02-24

**Authors:** Ram Das, Ramesh C. Dhiman, Deepali Savargaonkar, Anupkumar R. Anvikar, Neena Valecha

**Affiliations:** National Institute of Malaria Research (ICMR), Sector-8, Dwarka New Delhi, 110077 India

**Keywords:** Relapse malaria, *Plasmodium vivax*, Genotyping, Minisatellite marker

## Abstract

**Background:**

*Plasmodium vivax* malaria is a major public health problem in India. Control of vivax malaria is challenging due to various factors including relapse which increase the burden significantly. There is no well studied marker to differentiate relapse from reinfection. This creates hindrance in search for anti-relapse medicines. The genomic study of minisatellite can help in characterization of relapse and new infection of vivax malaria.

**Methods:**

Eighty-eight samples of *P. vivax* were collected from malaria clinic. All the 14 chromosomes of *P. vivax* were scanned for minisatellite marker by Tandem Repeat Finder software Version 4.07b. Minisatellite marker CH1T1M13779 from chromosome one was applied for genotyping in 88 samples of *P. vivax* including 2 recurrence cases.

**Results:**

Whole genome of *P. vivax* was scanned and found to have one hundred minisatellite markers. CH1T1M13779 minisatellite marker from chromosome-1 was used for amplification in 88 samples of *P. vivax.* Of 66 amplified samples, 14 alleles were found with varied allele frequency. The base size of 280 (13.63 %) 320 bp (13.63 %) and 300 bp (16.66 %) showed the predominant allele in the *P. vivax* population. Genotyping of two paired samples (day 0 and day relapse) could demonstrate the presence of relapse and reinfection.

**Conclusion:**

The CH1T1M13779 can be potential minisatellite marker which can be used to differentiate between relapse and new infection of *P. vivax* strain.

## Background

Malaria is a major global public health problem [1]. *Plasmodium vivax* is the most prevalent malaria parasite in Asia [2]. Relapsing nature of *P. vivax* poses a challenge for malaria elimination. Relapse rates of 5–40 % have been reported from India [3]. Relapses are known to occur at different time intervals, ranged from 1 month to 1 year. The long-term relapsing malaria has been reported from India [3–5]. It is difficult to differentiate relapse from reinfection and the existing methods have limitations.

Attempts have been made by using polymorphic markers and techniques for phylogenetic analyses in *P. vivax* like VNTR analysis [6], PCR–RFLP of *msp3* [7, 8], *msp1* [9, 10]. *csp* [9], *pvrbp*-*2* [11], *andgam*-*1* [12, 13]. Sequencing technique was also used for polymorphism analysis in genes of *msp1* [14], *msp*-*3α* [15], *msp*-*3β* [16], *msp5* [15, 17], *dpb* [18] and *vama1* [19]. Microsatellite markers were used for polymorphism analysis by GeneMapper [4, 20, 21].

An attempt was made to evaluate the utility of a newly designed minisatellite marker for differentiating relapse from reinfection.

## Methods

### Sample collection

**Fig. 1:**
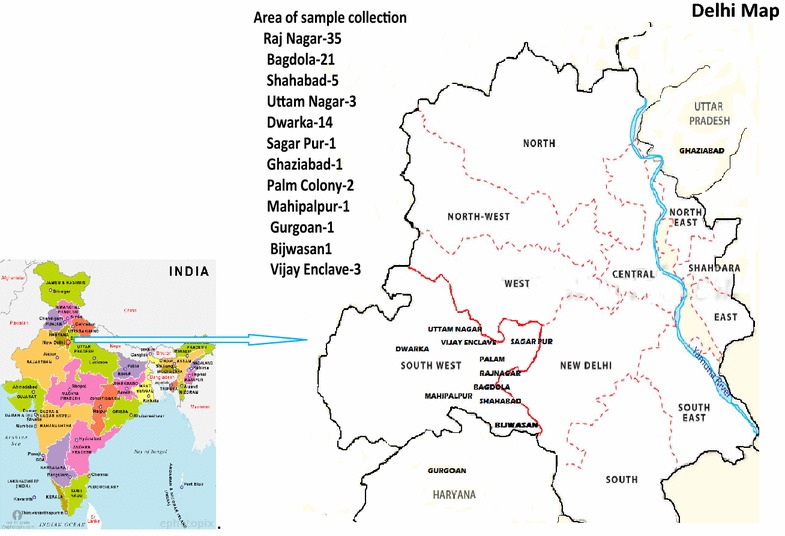
Area wise distribution of *P. vivax* samples

The blood samples from confirmed vivax malaria patients were collected by finger prick method aseptically from malaria clinic of National Institute of Malaria Research, New Delhi (Fig. 1). The diagnosis of malaria species was confirmed by microscopic method. Two to three drops of blood were also collected on 3 mm filter paper (Whatman International Ltd., Maidstone, UK) for DNA isolation. Altogether, 88 samples of *P*. *vivax* were collected. Patients were treated as per National Drug Policy of India [22]. Of 88 *P. vivax* cases, two reported back to malaria clinic after a lapse of nine and 11 months, while two patients from same family (father and son) reported on the same date. The ethics committee of National Institute of Malaria Research approved this study and written informed consent was obtained from the patients/guardians.

### Identification of minisatellite markers

All the 14 chromosomes of *P. vivax* were downloaded from National Center for Biotechnology (NCBI) and chromosome wise scanning for minisatellite markers was performed by Tandem Repeat Finder (TRF) software Version 4.07b [23]. One hundred minisatellite markers were identified by applying the parameters like period size (8–20), copy number (10–20), percent matches (90–100) and percent indels (0–2). Thirteen minisatellite markers were identified in the chromosome number one, of which three minisatellite marker’s primer sets were synthesized. Of three minisatellite markers, one minisatellite CH1T1M13779 which showed highly polymorphic nature was applied for amplification of 242 bp in 88 samples of *P. vivax.*

### DNA isolation and PCR amplification

The genomic DNA of *P. vivax* was extracted from the blood samples collected on the filter paper using QIAamp DNA Blood Mini Kit (Qiagen, Hilden, Germany) as per manufacturer’s instructions. PCR amplification of CH1T1M13779 was carried out in the final volume of 25 µl reaction contained 1× Buffer (50 mM KCl, 10 mM Tris, pH 8.3) 1.5 mM MgCl2, 0.2 mM dNTPs, 10 pmol of each primer forward (F-5′-GCATCATAATGGGTAAAG-3′) and reverse (R-5′-TCTCAATCACTGCAACAA-3′), 0.2 µM of dNTPs, 0.75 U of Taq DNA polymerase and 2 µl of genomic DNA. The thermal cycler parameter: initial denaturation at 94 °C for 5 min followed by 45 cycles denaturation at 94 °C for 1 min, annealing at 46 °C for 1 min, extension at 72 °C for 1.30 min and final extension at 72 °C for 10 min. The amplified PCR products and control were resolved on 2 % of metaphore agarose gel. The gel was stained with ethidium bromide and visualised under the UV light.

## Results

### Genome scanning for minisatellite marker and validation in clinical samples of *Plasmodium vivax*

**Fig. 2:**
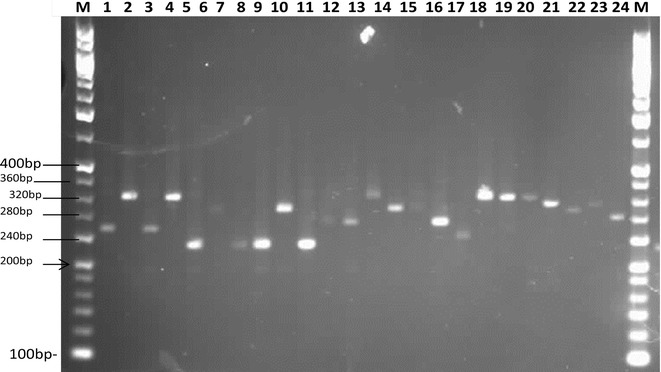
PCR amplification of minisatellite marker showing base size polymorphisms (Sample: *Lane 1 to 24*; M: 20 bp DNA ladder)

**Fig. 3:**
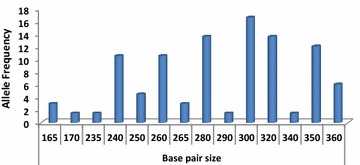
Allele frequency and base size polymorphism in the *Plasmodium vivax* population

One hundred minisatellite markers were identified in the whole genome sequence of *P. vivax.* Of 88 samples, 66 were amplified and showed 14 alleles with varied allele frequency (Fig. 2). Period size 13 (AGCAAAGGTAGGC) and copy number 10.5 showed 14 alleles in the 66 samples. The allelic frequencies were 165 bp in 3.03 %, 170 bp in 1.51, 235 bp in 1.51, 240 bp in 10.60, 250 bp in 4.54, 260 bp in 10.60, 265 bp in 3.03, 280 bp in 13.63, 290 bp in 1.51, 300 bp in 16.66, 320 bp in 13.63, 340 bp in 1.51, 350 bp in 12.12 and 360 bp in 6.00 % of the *P. vivax* population (Fig. 3). The base size of 280 bp in 13.63 %, 320 bp in 13.63 % and 300 bp in 16.66 % showed the predominant allele in *P. vivax* population (Table 1).

**Table 1:** Area wise distribution of patient samples and allele frequency in *P. vivax* population

Base size	Area wise distribution *P. vivax* samples												Total no. sample	Allele frequency (%)
	RN	BD	SB	UN	D	SP	G	PC	MP	GU	B	VE		
165	1	1											2	3.03
170					1								1	1.51
235	1												1	1.51
240	1		2		3							1	7	10.6
250	1								1			1	3	4.54
260	5	2											7	10.6
265	1					1							2	3.03
280	4	1		2	2								9	13.63
290	1												1	1.51
300	5	3	1		1		1						11	16.66
320	6				1					1	1		9	13.63
340								1					1	1.51
350	1	3		1	2			1					8	12.12
360	3				1								4	6.06
Total	30	10	3	3	11	1	1	2	1	1	1	2	66	

*RN* Raj nagar, *B* Bagdola, *SH* Shah bad, *UN* Uttam nagar, *D* Dwarka, *SP* Sagar pur, *G* Ghaziabad, *PC *Palm colony, *MP* Mahipal pur, *GU* Gungaon, *B *Bijwasan, *VE* Vijay enclave

### Validation of minisatellite in case control study

**Fig. 4:**
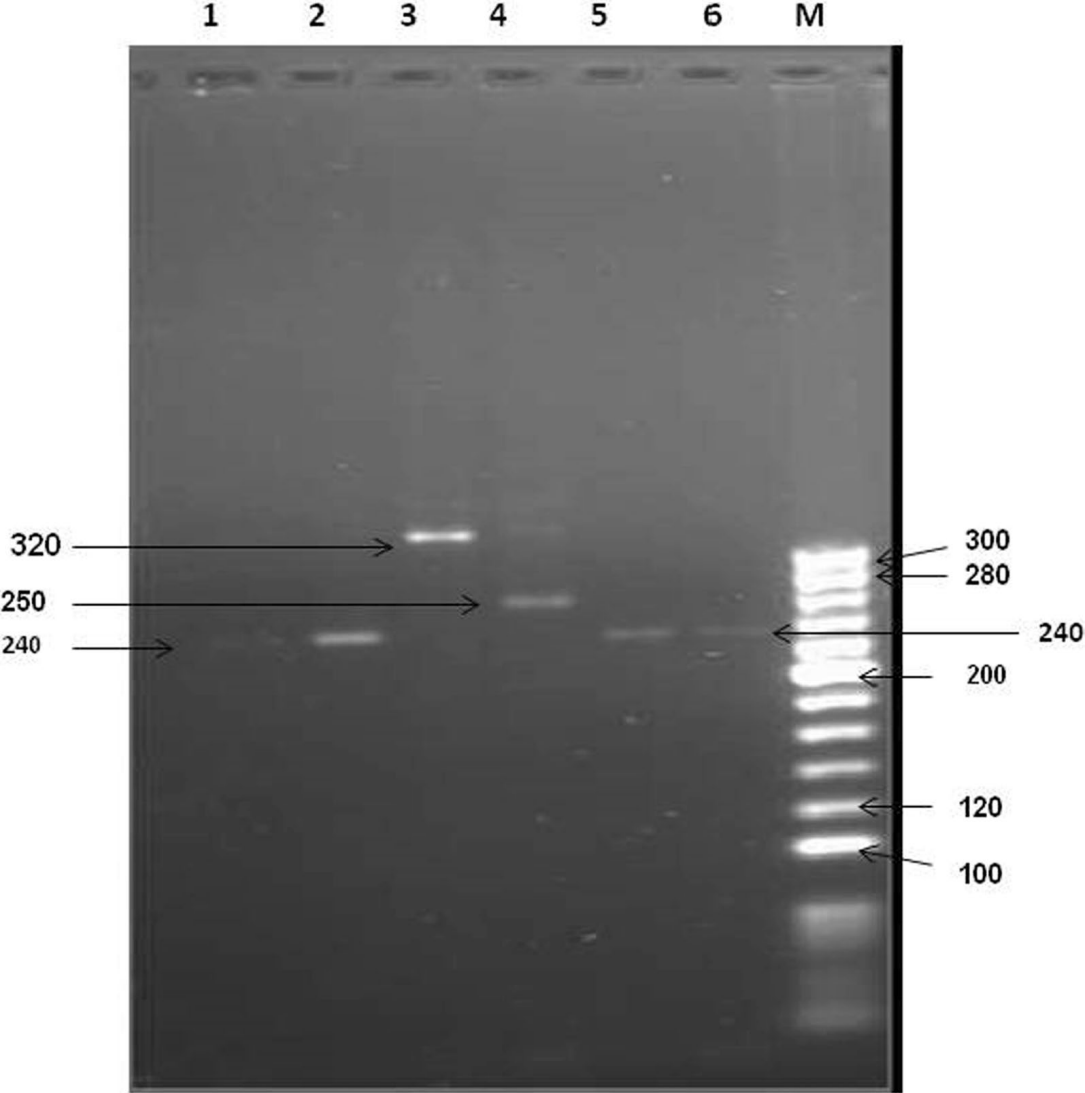
*Lane: 1,2* same allele (relapse), *Lane: 3,4* different allele (new infection), *Lane: 5,6* same allele (father and son), Lane M: 20 bp ladder marker

Minisatellite marker CH1T1M13779 was used for case study in relapse, new infection and transmission pattern of *P. vivax*. Genotyping of two samples collected on day 0 and after 9 months from one patient from Dwarka region showed the identical genotype (Fig. 4, Lane 1 and 2). Both the samples from this patient showed same strain of *P. vivax* which caused the relapse after 9 months (Table 2).

**Table 2:** Minisatellite marker CH1T1M13779 validate in 4 case control studies

Case no.	Blood sample collection		Allele	Interpretation
	Day 0 (zero)	After day 0 (zero)		
I	I	II (after 9 months)	Similar (240 bp)	Relapse
II	I	II (after 11 months)	Different (250 and 320 bp)	New-infection
III and IV	I		Similar (240 bp)	Transmission of same parasite in father and son

Genotyping of another patient based on samples collected on day 0 and after a lapse of 11 months from Vijay Enclave showed two different genotypes (Fig. 4, Lane 3 and 4). Different genotype from same patient showed new infection due to different strain of *P. vivax* (Table 2). Genotyping of two patients from same family (father and son) who reported at the clinic on same day from Shahabad showed identical genotype (Fig. 4, Lane 5 and 6). The identical genotype of both the patients showed transmission of one *P. vivax* strain in the family (Table 2).

## Discussion

The control and elimination of *P. vivax* is a major challenge due to the relapses. Relapses need to be differentiated from reinfection for knowing true efficacy of anti-relapse medicines during clinical trials. Current techniques to differentiate relapse from reinfections have limitations. The technique PCR–RFLP is used for allelic discrimination and polymorphism analysis in the genes such as *Pvmsp3α,msp1* [24, 25], sequencing of microsatellite [4], capillary electrophoresis-based heteroduplex tracking assay [25, 26]. Recently, deep sequencing technique has been used for differentiation of relapse and reinfection [27].

These techniques are time consuming, expensive and need well established molecular laboratory. In the present study, whole genome of *P. vivax* was scanned and designed the simple, rapid, cost effective technique for differentiation of relapse and reinfection. The newly designed minisatellite marker CH1T1M13779 is highly polymorphic in nature and showed 14 alleles with varied allele frequency. The base size of 280 bp (13.63 %), 320 bp (13.63 %) and 300 bp (16.66 %) were predominant allele in *P. vivax* population.

The paired samples from one patient (day 0 and day recurrence at nine months) were analysed by minisatellite marker CH1T1M13779. Samples from another patient collected on day 0 and 11 months showed two different genotypes demonstrating that it was new infection due to different strain of *P. vivax*. Thus, this minisatellite marker could help to differentiate the relapse from new infection of *P. vivax*. Genotyping data of *P. vivax* by minisatellite marker supports the hypothesis of Kim et al. [10] and Koepfli et al. [28].

Genetic diversity of *P. vivax* has significant impact on malaria transmission [29]. The identical genotype of *P. vivax* in a father and son showed that transmission occurred due to same strain. Thus, the marker may also be helpful in identifying the origin and transmission of parasite in family or locality. Genotyping by the minisatellite marker can also help to differentiate *P. vivax* relapse from reinfection and thus help to determine efficacy of antirelapse medicines. This preliminary data shows that there is need to further strengthen data using more number of minisatellite markers in more paired *P. vivax* sample for statistical conclusions. This technique is rapid and cost effective as compared to the PCR–RFLP, sequencing, gene scanning and heteroduplex tracking assay.

Further, the study of polyclonal infections of *P. vivax* by the capillary electrophoresis-based heteroduplex tracking assay [25] and deep sequencing for detection of genetic signatures can generate important data that could be useful in developing antirelapse medicine trials.

## Conclusion

Study provided preliminary evidence of identifying the relapse and new infection by newly designed minisatellite marker CH1T1M13779. Further studies are needed to validate the same.
